# Fatal familial insomnia with abnormal signals on routine MRI: a case report and literature review

**DOI:** 10.1186/s12883-017-0886-2

**Published:** 2017-05-26

**Authors:** Tingting Lu, Yuhang Pan, Lisheng Peng, Feng Qin, Xiaobo Sun, Zhengqi Lu, Wei Qiu

**Affiliations:** 1 Department of Neurology, the Third Affiliated Hospital of Sun yat-sen University, Guangzhou, China; 2 Department of Pathology, the Third Affiliated Hospital of Sun yat-sen University, Guangzhou, China; 3 Department of Neurosurgery, the Third Affiliated Hospital of Sun yat-sen University, Guangzhou, China

**Keywords:** Fatal familial insomnia, Cadasil, Prion, Dementia, Magnetic resonance imaging

## Abstract

**Background:**

Fatal familial insomnia (FFI) is a rare autosomal dominant disease caused by the PRNP D178N/129 M mutation. Routine brain CT and MRI usually reveal non-specific features. We report a patient with FFI presenting with diffuse abnormal signals on MRI, later confirmed as combined with cerebral autosomal dominant arteriopathy with subcortical infarcts and leukoencephalopathy (CADASIL).

**Case presentation:**

The patient was a 58-year-old female, whose main clinical manifestations were insomnia, movement disorders, autonomic hyperactivity and mental deterioration. The patient also suffered a typical episode of transient global amnesia. MRI indicated a diffuse white matter abnormality and microbleeding on the susceptibility-weighted imaging. On biopsy, the brain tissue sections showed spongiform changes with gliosis, neuronal degeneration, and prion protein deposition in a portion of the neurons. In addition, arteriosclerosis was prominent. Transmission electron microscopy showed osmiophilic particle deposition in the matrix of medial smooth muscle cells. Gene sequencing confirmed a diagnosis of FFI with CADASIL.

**Conclusions:**

This case is a compelling example that even with evidence of leukoencephalopathy, prion disease should be an important differential diagnosis of rapidly progressive dementia and related diseases. In cases of genetic diseases with atypical manifestations, the coexistence of two or even more diseases should be considered as a possible explanation.

## Background

Fatal familial insomnia (FFI) is a rare autosomal dominant disease caused by the prion protein gene (PRNP) D178N/129 M mutation [1]. Since the initial report by Lugaresi et al. in 1986 [2], more than 100 FFI pedigrees have been reported around the globe. Consistent with other prion diseases, routine brain CT and MRI usually reveal non-specific features [3]. We report an FFI patient presenting with diffuse abnormal signals in the white matter, basal ganglia and thalamus on MRI as a result of small vessel disease, which was later confirmed as combined with cerebral autosomal dominant arteriopathy with subcortical infarcts and leukoencephalopathy (CADASIL).

## Case presentation

**Fig. 1:**
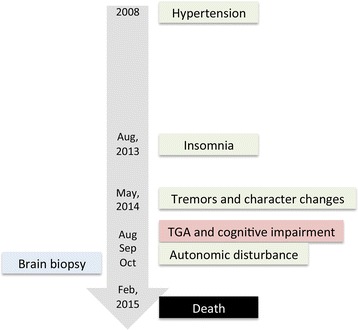
The timeline of the patient’s history

**Fig. 2:**
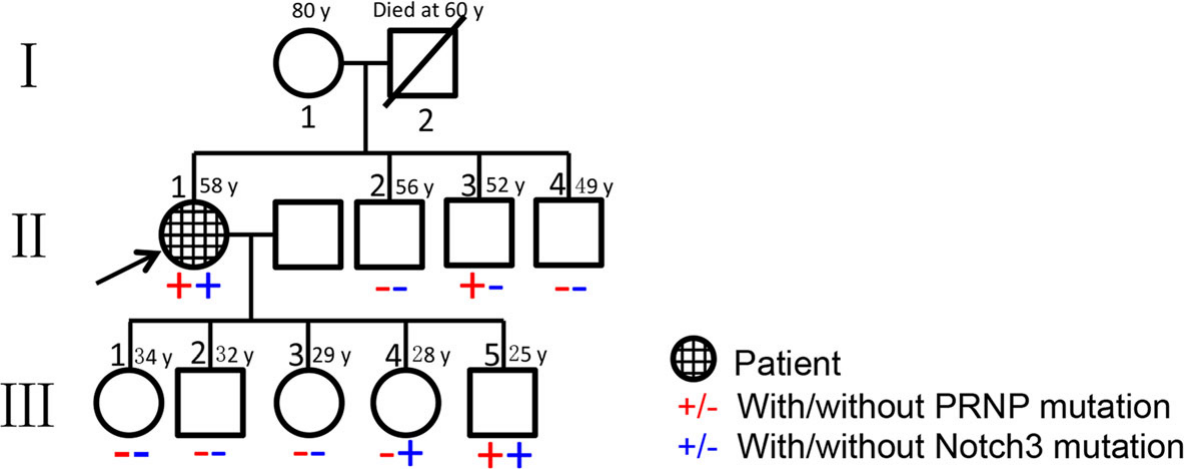
The patient’s pedigree. The arrow indicates the proband (our patient). The father of our patient (I-2) had a history of hypertension and died of stroke. A son of our patient (III-5) developed hypertension at the age of 27

A 58-year-old female was hospitalized for sleep disturbance and abnormal behaviors in October 2014. The reduction in sleep time and early awakening started in the middle of 2013. In May 2014, other family members noticed that the patient’s character had changed. She had an apathetic appearance and postural tremors in both hands. Snoring while sleeping was also noticed. In August, the patient was found to have unconscious talking, large amplitude limb movements, and shrill laryngeal sounds while inhaling during sleep. Occasionally, the patient could not fall asleep all night. During the daytime, intermittent peculiar oneiric behaviors were noticed. She also suffered a typical period of transient global amnesia (TGA), which lasted for 6 h. The patient was unable to do housework. However, she was still able to recognize all her family members and recall long-term memories with relative deterioration of her short-term memory. Uroschesis and constipation with obvious hyperhidrosis developed in September. The patient had a history of hypertension for approximately 5–6 years and discontinued the medicine 7 months before admission (Fig. 1). Her father, who died in his 60s due to a cerebrovascular event, was also found to have had hypertension. The pedigree is summarized in Fig. 2.

**Fig. 3:**
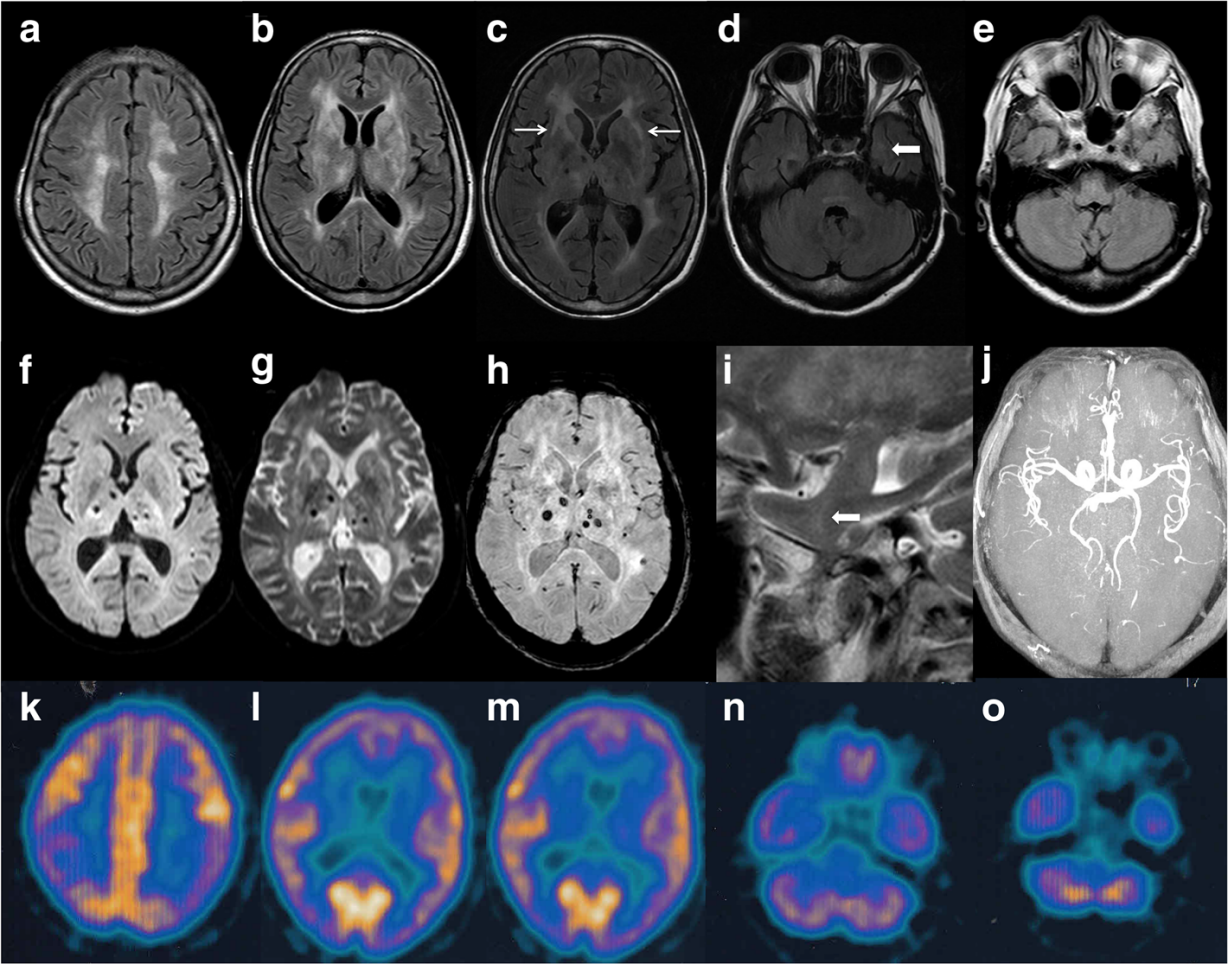
The patient’s cranial imaging findings. **a-j** Magnetic resonance imaging showed abnormal signals in the bilateral frontoparietal subcortical area, periventricular region of the lateral ventricle, basal ganglia, and dorsal thalamus. The bilateral external capsules (**c**) and left temporal pole **d** and **i** were affected as indicated by *arrows*. h, SWI revealed multiple hemosiderin depositions or minor hemorrhage lesions in the bilateral basal ganglia, dorsal thalamus, and right parietal lobe. **j**, MRA indicated reduced distal branches of the cerebral arteries. **k-o**, 18Fluoro-deoxy-glucose-PET/CT showed reduced glucose metabolism in the bilateral lobes, basal ganglia, and thalamus

Upon admission, the patient was fully conscious but showed significant deficits in temporal and spatial orientation, recent memory, and calculation abilities. The patient had moderate bulbar palsy, increased muscle tone with normal muscle strength in all four limbs and impaired stability and accuracy in coordinating movements. Her tendon reflexes were hyperactive except for the ankle reflex. Pathologic reflexes were positive on both sides. Myoclonus was not noticed. The patient had a body temperature of 36.7–37.3 °C, a heart rate of 82–127 bpm, a blood pressure of 127–167/63–97 mmHg and persistent hyponatremia (125–132 mmol/L) and hypochloremia (82–92 mmol/L). The glycosylated hemoglobin (GHbA1c) level was 6.5% (6.0% two months ago), and the blood homocysteine level was 22.55 μmol/L. Dynamic electrocardiogram indicated low heart rate variability. Cerebrospinal fluid (CSF) testing showed a mildly elevated protein concentration (0.53 g/L). The CSF was positive for the 14–3-3 protein. Neither periodic spike discharges nor sleeping activity was found on the electroencephalogram. Brain MRI indicated diffuse abnormal signal in the bilateral frontoparietal subcortical areas, the periventricular area of the lateral ventricles, the basal ganglia, and the dorsal thalamus with external capsule and temporal pole involvement, along with malacia in the right centrum semiovale. The lesions showed slight hypersignal on diffusion-weighted imaging (DWI) and low signal on apparent diffusion coefficient (ADC). Susceptibility-weighted imaging (SWI) revealed multiple hemosiderin depositions or minor hemorrhage lesions in the bilateral basal ganglia, dorsal thalamus, and right parietal lobe. Magnetic resonance angiography (MRA) revealed reduced branches of the cerebral arteries. ^18^Fluoro-deoxy-glucose-PET/CT showed hypometabolism in the bilateral parietal lobes, subcortical areas, basal ganglia, and thalamus (Fig. 3).

**Fig. 4:**
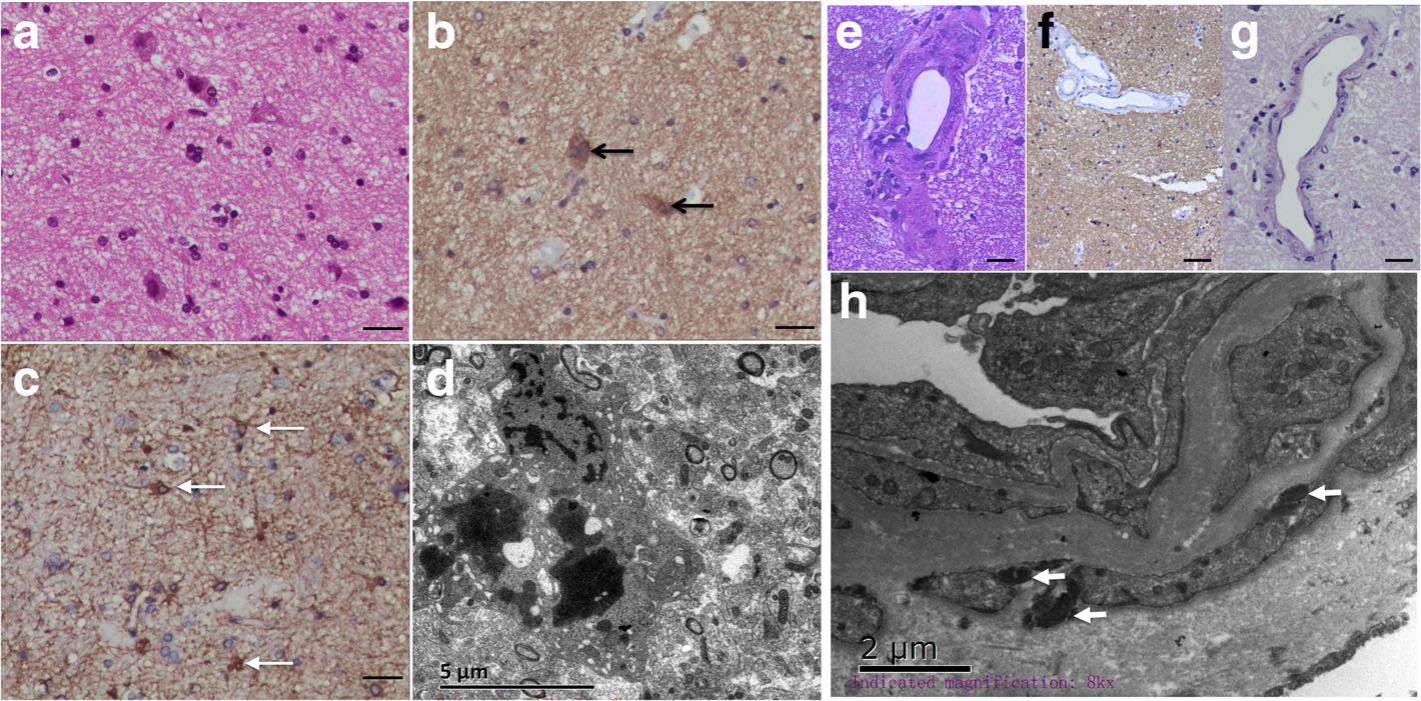
Pathological results of biopsy from the head of the right caudate nucleus. **a** Hematoxylin and eosin staining showed neuronal degeneration, as well as spongiform changes in the brain tissue. **b** A portion of neurons were stained by PRNP antibody (*black arrows*). **c** Gliosis was observed after staining with GFAP antibody (*white arrows*). **d** Degenerated neurons were observed under electron microscope. **e** Thickening of the walls and hyalinization of small blood vessels in the brain tissue were apparent. **f** Immunohistological staining showed no perivascular PRNP protein deposition. **g** Congo red staining of the small cerebral vessels was negative. Bar = 25 μm. **h** Multiple granular osmiophilic material depositions on the surface of vascular smooth muscle cells (*white arrows*) were observed under transmission electron microscopy examinations

**Fig. 5:**
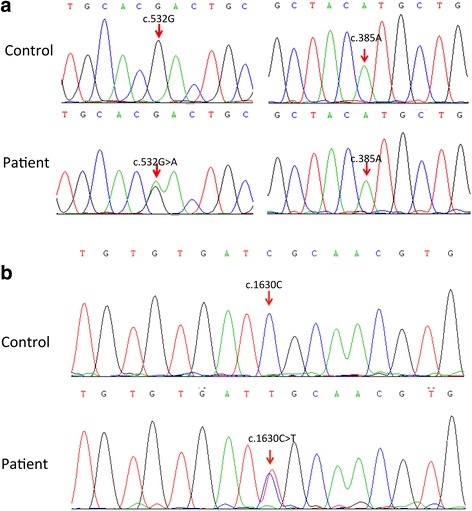
The chromatograms of sequencing results. **a** PRNP gene sequencing revealed a heterogeneous D178N mutation (sequence variant. 532G > A) in combination with the polymorphism 129 M (sequence variant: (*c*). 385A). **b** The sequencing result of Notch3 indicated a heterozygous (*c*) 1630C > T (p. Arg544Cys) mutation

For further diagnosis, the patient underwent a CT-guided stereotactic brain biopsy, and tissues were obtained from the head of the right caudate nucleus. Spongiform encephalopathy was identified. Neuronal degeneration was observed under both light and transmission electron microscope. Astrogliosis was also prominent. A portion of the neurons were stained by the PrP antibody. However, arteriosclerosis was also prominent. Transmission electron microscopy revealed visible osmiophilic particle deposition in the matrix of the medial smooth muscle in the cerebral arterioles (Fig. 4). Sequencing of the PRNP gene revealed the D178N/129 M mutation. Sequencing of the Notch3 gene revealed a mutation of c.1630 C > T (p. Arg544Cys), a reported pathogenic mutation [4] (Fig. 5). The mutations in PRNP and Notch3 were validated using Sanger sequencing in the patient’s three brothers and five children (Fig. 2). Exome sequencing of previously identified 4811 genes was performed with a MiSeq sequencer (Illumina Inc., USA); no other compelling mutation was identified. The final diagnosis was FFI combined with CADASIL.

At the end of October, the patient showed aggravated tremor and crocidismus. She could easily fall on her back. Most of the time, she was in a semi-asleep and semi-awake hazy state. The symptoms gradually worsened. The patient died in February 2015, after a total disease duration of 20 months. Before her death, the patient exhibited severe mental disorders, total sleep deprivation, significantly increased blood pressure and a heart rate continuously above 140 bpm.

Differing from Creutzfeldt-Jakob disease (CJD), which mainly affects the cortex, the deposition of abnormal PrP^SC^ in FFI selectively affects the dorsomedial and anteroventral thalamic nuclei and the inferior olivary nucleus of the brainstem, whereas cortical areas such as the cingulate, frontal, and parietal cortexes are affected only in the late stages. The clinical characteristics are consistent with the affected brain areas [5]. Sleep disorders are the earliest and most prominent symptoms, including progressively shortened total sleep time, loss of sleep spindles and δ waves, significantly reduced durations of rapid eye movement sleep and slow wave sleep, as well as automatic behaviors, complex hallucinations and vivid dreams during sleep, that cannot be relieved by common hypnotics. FFI patients also have prominent autonomic dysfunctions. Endocrine changes, movement disorders and cognitive dysfunction occur in the relatively late stages. The clinical manifestations in this patient included insomnia, movement disorders, autonomic hyperactivity, and mental deterioration. Insomnia combined with laryngeal sounds during sleep was the most prominent symptom. These manifestations fit the characteristics of FFI. The diagnosis was confirmed using PRNP gene sequencing.

However, there were atypical features in our case. The typical pathological features of FFI include severe atrophy of the mediodorsal and anteroventral thalamic nuclei and of the inferior olives in the absence of spongiform changes [6]. Due to the relatively low PrP^Sc^ accumulation, immunohistochemistry on paraffin sections usually fails to detect PrP^Sc^ in any brain region except for cases with a relatively long disease duration [7]. However, the brain tissue from the head of the right caudate nucleus in our case showed typical neurodegeneration, gliosis and PrP^Sc^ deposition, indicating a larger distribution of the affected brain areas. Moreover, spongiform changes were significant. Correlating to the classic areas of PrP^Sc^ deposition, PET typically indicates hypometabolism predominantly in the thalamus and cingulate cortex in FFI [6, 8]. The hypometabolic areas in our case broadly involved the parietal lobes and subcortical areas. Second, the CSF is usually negative for 14–3-3 protein in FFI. However, our patient had increased levels of this protein. Phenotype–genotype correlation studies have revealed considerable clinical and pathological variability in patients with the D178N/129 M mutation, suggesting a continuous spectrum between the FFI and CJD phenotypes [9, 10]. Interestingly, the symptoms of our patient were consistent with FFI. However, the presence of 14–3-3 protein, spongiform changes in the tissue sections and the hypometabolic area on PET showed features of CJD.

Finally and importantly, the patient also suffered a typical episode of TGA. The MRI indicated diffuse white matter abnormalities, involving the bilateral thalamus, basal ganglia, external capsule and temporal pole, a reduction of the branches of the cerebral arteries on MRA and microbleeding on SWI. These features indicated the involvement of the vascular system, particularly the small cerebral vessels. The small vessel changes on biopsy confirmed arteriosclerosis. A stop mutation at codon 145 of PRNP can cause the deposition of PrP amyloid in cerebral vessels [11]. However, no evidence of amyloid angiopathy, including amyloid angiopathy caused by prions, was found in the brain sections. The arteriosclerosis was first speculated to be a consequence of hypertension caused by the higher background and stimulated sympathetic activity in FFI [1, 2, 12]. However, electron microscopy and Notch3 gene sequencing confirmed CADASIL.

The typical brain MRI feature in prion diseases is the hyperintense signal along the cerebral cortex and subcortical gray matter on DWI, whereas routine T1- and T2-weighted imaging reveals nonspecific cerebral and cerebellar atrophy [3, 13]. FFI patients may show hypersignal in the basal ganglia and other gray matter areas on DWI [3]. Abnormal signals on routine MRI sequences have only been reported in two other cases of fatal insomnia. In the case of a patient with sporadic fatal insomnia with a medical history of diabetes mellitus and hypertension, non-specific microvascular ischemic white matter disease was noticed on fluid-attenuated inversion recovery (FLAIR) images [14]. In another case of FFI, posterior reversible encephalopathy syndrome was observed [15]. Although concomitant diseases could not be ruled out, abnormal signals on routine MRI in the two cases could be explained by the vascular insults caused by hypertension and other autonomic system dysfunction. To support this view, hypoperfusion was observed on perfusion weighted imaging (3D–ASL) in a case of prion disease with the D178N mutation [3]. The observed coexistence of FFI and CADASIL in our case has not been previously reported and contributed to extremely rare clinical and MRI manifestations and difficulty in diagnosis. It is a compelling example that prion disease should be an important differential diagnosis of rapidly progressive mental deterioration and should not be excluded, even in patients with MRI features of leukoencephalopathy.

The two responsible genes, PRNP and Notch3, are located on chromosomes 20 and 19, respectively. There was no proved functional interaction between the two genes. So, it is unlikely that the two genes are linked. This could also be indicated by the Sanger sequencing results of the family members (Fig. 2). The exact reason for the coexistence of the two mutations is unknown. It could be coincidental or could be the result of a mechanism not yet elucidated. An open issue in our case is the family history of hypertension. A proper explanation was that the hypertension was secondary to FFI or CADASIL, as previously reported [2, 16]. Though previously rarely noticed, the coexistence of two or even more mutations should be listed as a possible explanation in cases of genetic diseases with atypical or complex manifestations. After the clinical application of next generation sequencing of whole genomes and exomes, more illustrations of this situation could be identified [17–20]. In a few recent clinical whole exome sequencing cohorts, 1.4–7.2% patients were diagnosed with multiple genetic diseases [21, 22]. These results challenge the diagnostic principle that medical practitioners should try to identify the most evident single explanation on the basis of history and physical examination. Whole genome and exome sequencing would be powerful tools for the precise diagnosis of hereditary diseases with complex clinical presentations.

## Conclusions

We reported a case involving the coexistence of FFI and CADASIL, which are two genetic disorders that mainly affect the brain. The underlying mechanism of this coexistence is not clear. It is an indication that prion diseases cannot be ruled out in cases of leukoencephalopathy. Furthermore, in cases of genetic diseases with atypical manifestations, the coexistence of two or even more diseases should be listed as a possible explanation.

## Abbreviations

ADC: Apparent diffusion coefficient
CADASIL: Cerebral autosomal dominant arteriopathy with subcortical infarcts and leukoencephalopathy
CJD: Creutzfeldt-Jakob disease
CSF: Cerebrospinal fluid
CT: Computer tomography
DWI: Diffusion weighted image
FFI: Fatal familia insomnia
FLAIR: Fluid-attenuated inversion recovery
GHbA1c: Glycosylated hemoglobin
MRA: Magnetic resonance angiography
MRI: Magnetic resonance imaging
SWI: Susceptibility weighted imaging
TGA: Transient global amnesia
